# The First Observation of the Filamentous Fungus *Neurospora crassa* Growing in the Roots of the Grass *Brachypodium distachyon*

**DOI:** 10.3390/jof10070487

**Published:** 2024-07-14

**Authors:** Krisztina Kollath-Leiß, Urska Repnik, Hannes Winter, Heinrich Winkelmann, Anna Sophia Freund, Frank Kempken

**Affiliations:** 1 Abt. für Botanische Genetik und Molekularbiologie, Botanisches Institut und Botanischer Garten, Christian-Albrechts-Universität, 24098 Kiel, Germany; hwinter@bot.uni-kiel.de (H.W.); annasophiefreund@web.de (A.S.F.); 2 Zentrale Mikroskopie, Christian-Albrechts-Universität, 24118 Kiel, Germany; urepnik@bio.uni-kiel.de

**Keywords:** *Neurospora crassa*, *Brachypodium distachyon*, endophytic lifestyle, plant–fungus interaction

## Abstract

The model organism *Neurospora crassa* has been cultivated in laboratories since the 1920s and its saprotrophic lifestyle has been established for decades. However, beyond their role as saprotrophs, fungi engage in intricate relationships with plants, showcasing diverse connections ranging from mutualistic to pathogenic. Although *N. crassa* has been extensively investigated under laboratory conditions, its ecological characteristics remain largely unknown. In contrast, *Brachypodium distachyon*, a sweet grass closely related to significant crops, demonstrates remarkable ecological flexibility and participates in a variety of fungal interactions, encompassing both mutualistic and harmful associations. Through a comprehensive microscopic analysis using electron, fluorescence, and confocal laser scanning microscopy, we discovered a novel endophytic interaction between *N. crassa* and *B. distachyon* roots, where fungal hyphae not only thrive in the apoplastic space and vascular bundle but also may colonize plant root cells. This new and so far hidden trait of one of the most important fungal model organisms greatly enhances our view of *N. crassa*, opening new perspectives concerning the fungus‘ ecological role. In addition, we present a new tool for studying plant–fungus interspecies communication, combining two well-established model systems, which improves our possibilities of experimental design on the molecular level.

## 1. Introduction

The fungus *Neurospora crassa* is a filamentous ascomycete of the Sordariaceae family. Since its introduction to laboratories in the 1920s, *N. crassa* has been employed as a model eukaryotic multicellular organism for genetics, developmental biology, and molecular biology [1,2]. This fungus has been instrumental in working out important genetic principles. Most famous is the use of *N. crassa* mutants for the one-gene-one-enzyme hypothesis [3]. Other achievements include DNA methylation-mediated epigenetic control or repeat-induced point mutation mechanisms to name only a few [4,5]. The availability of a whole genome deletion library, a bright spectrum of established genetic tools, and its rapid vegetative growth and ease of sexual mating made *N. crassa* a versatile fungal model organism [6,7,8,9].

Despite this extensive research on *N. crassa*, little is known about its natural populations and ecology [10,11,12]. The fungus is assumed to be saprotrophic. Wild populations have been isolated in parts of India, Pakistan, Middle Africa, South America, and the USA (Louisiana and Texas) from grass, soil, wild sugarcane, cane stubble, and wood [13,14]. A single publication also reported the endophytic growth of *N. crassa* in pine trees [15], which was never confirmed independently.

We set out to investigate a potential interaction of *N. crassa*, employing *Brachypodium distachyon*, which is a sweet grass and thus closely related to important crop plants like barley, wheat, or sugarcane [16,17,18]. The species shows a wide distribution and it is highly adaptable in terms of its ecological environment [19].

Plant tissues offer a niche for diverse microorganisms, which can develop a wide range of interactions from symbiotic to parasitic with the host plant. Endophytic bacteria and fungi can colonize the plant’s inter- or intracellular place asymptomatically and establish a mutualistic relationship with the host [20,21,22]. Endophytic fungi (EF) are ubiquitous in the plant kingdom [23], and their origin dates back to the evolution of land plants [24]. To date, only appr. 150,000 of the estimated 3 million endophytic fungal species have been classified [25]. EF mostly belong to the phyla Asco- and Basidiomycota [26]. They colonize roots, stems, or leaves of a wide range of plant species [27]. The interaction is beneficial for both counterparts, as it enhances the plant’s competitive abilities and improves the fungus’ nutrition supply. However, the EF can switch to a parasitic lifestyle according to stress conditions, nutrition supply, microbe titer, and the genotypes of the interacting organisms [28]. Therefore, the maintenance of an enduring, stable endophytic interaction requires a strategy for a mutualistic balance between host defense and fungal virulence under the challenging conditions of a complex microbiome [29]. EF, for example, produce a high diversity of primary and secondary metabolites, enzymes, and volatile compounds as a direct strategy to overcome the host’s immune system [23]. Especially the production of novel secondary metabolites made EF interesting to investigate from the application perspective [26]. Despite the large number of studies conducted on EF, the molecular mechanisms for mutual adaptation after fungal infection are still unclear [22,30].

Remarkably, our research provides strong evidence that the saprotrophic fungus *N. crassa* can grow in the roots of the grass *B. distachyon* in an endophytic manner, providing a novel and easily cultivable interaction system for microbiological investigations of endophytic interactions.

## 2. Materials and Methods

### 2.1. Strains, Growth Condition

The following *N. crassa* strains used in this study were provided by the Fungal Genetics Stock Center (FGSC; Kansas City, MO, USA): FGSC #4200 (wild type, mat A) and FGSC #9518 (his-3+::Pccg-1-hH1+-sgfp+, mat A) [31]. Fungi were cultivated on Vogel’s minimal medium [VMM, [32]]. For the co-cultivation of *N. crassa* with plants, solid or liquid Vogel’s minimal medium with reduced sucrose content (0.2%) was used. For the first investigations, an EcoFAB 1.0 device with a liquid medium was used [33]. EcoFABs are fabricated ecosystems that allow for controlled plant–microbe interaction studies under sterile conditions. We used this device for our first microscopical studies to investigate the interaction of the hyphae with the root surface.

We first decided to investigate the formerly reported interaction of the fungus with *Pinus sylvestris.* We also tried co-cultivation with mono- and dicotyledonous species, which are easier to cultivate, namely *Arabidopsis thaliana* and *Zea mays*. Finally, we decided to use the model organism *Brachypodium distachyon*, which develops diverse interactions with microbiota. *Zea mays* and *Brachypodium distachyon* TR7a wild-type caryopses as well as *A. thaliana Col-0* and *Pinus sylvestris* wild-type seeds were provided by the Botanical Garden of the CAU zu Kiel (Kiel, Germany). All seeds and caryopses were surface-sterilized according to Weigel and Glazebrook [34] before cultivation. In the case of *B. distachyon,* the lemma and palea of the caryopses have been removed manually prior to surface sterilization. After surface sterilization, Pinus seedlings were vernalized for 7 days at 7 °C in the dark, and *B. distachyon* plants were vernalized for 48 h at 7 °C in the dark. All plants were cultivated under controlled sterile conditions in a climate chamber (22 °C, 60% humidity, 16 h light, and 8 h dark). Germlings that were 7–10 days old were inoculated with *N. crassa*. For this, macro-conidiospores were transferred from a mycelium by a loop and plated at the roots’ vicinity under sterile conditions. Inoculated plants were cultivated under sterile conditions in a climate chamber (25 °C, long day of 16 h/8 h).

### 2.2. Fixation, Sectioning, and Staining

For analyses by confocal laser scanning microscopy (CLSM), infected plants were immediately fixed in 4% (*w*/*v*) paraformaldehyde for 30 min in a vacuum. After 15 min, the fixing solution was replaced. Subsequently, the fixed samples were washed in 100 mM Tris/HCl, pH 8.8, with 0.1% (*v*/*v*) glycerol and stored overnight at 7 °C. The root part of the samples was then divided into ca. 1 cm long sections (designated as “root tip”, “S1”, and “S2”) embedded in 4% (*w*/*v*) agarose. Longitudinal sections measuring at 60–120 µm were prepared by using a Zeiss HYRAX V50 vibratome (Zeiss, Oberkochen, Germany).

For paraffin embedding, 1 cm long root terminals were cut into 2 mm segments and fixed with 4% paraformaldehyde in 200 mM HEPES, pH 7.4, for several days. Samples were dehydrated by a rising ethanol series (50-70-80-90-96-100-100%), followed by 2 steps of 100% acetone. For clearing, samples were incubated in an acetone and xylol 1:1 mixture, and three steps of 100% xylol, including one overnight. Next, samples were incubated in Paraplast Plus at 60 °C overnight and embedded the next day. Blocks were cut into 7 μm sections using a Leica RM2200 rotary microtome (Leica, Wetzlar, Germany), stretched on a warm water bath, transferred onto adhesion slides from Superfrost^®^ Plus, and stained with hematoxylin and eosin using a standard protocol.

### 2.3. Microscopy

For the first analyses of the interaction, an epi-fluorescence microscope (ECLIPSE Ci, Nikon, Tokyo, Japan) equipped with a GigE camera (Imaging Source, Bremen, Germany) was used. Epi-fluorescence filter block GFP (longpass, excitation: 480/40 nm, dichroic mirror: 505 nm, barrier filter: 510 nm) was used for green fluorescence detection. Images were analyzed using Nikon software NIS Elements D basic (https://www.microscope.healthcare.nikon.com/de_EU/products/software/nis-elements, accessed on 1 July 2024).

Confocal fluorescence analyses were performed using the confocal laser scanning microscopes Leica TCS SP5 (Leica) equipped with an HCX PL Apo 63×/1.2 W objective (Leica) or—especially for overview images—Zeiss LSM 900 (Zeiss) using 10× (NA 0.45 M27), 20× (NA0.8 M27), and 40× (NA 1.45 oil DIC (UV) VIS-IR M27) Plan-Achromat (Zeiss) objectives. Fusion constructs with eGFP were excited at 488 nm, and emission was detected at 500–550 nm. Images were analyzed with Leica LAS AF Lite or Zeiss ZEN3.2 blue edition software, respectively.

Stained paraffin sections were imaged with a Zeiss Primostar upright microscope (Zeiss) equipped with an Axiocam 105 color camera and ZEN 3.2 software, using 10× and 40× Plan-Achromat objectives.

### 2.4. Electron Microscopy

For the electron microscopic analysis, 1 cm long root terminals were cut into 2 mm segments and fixed with 1% glutaraldehyde in 200 mM HEPES, pH 7.4, for several days. For scanning electron microscopy (SEM), segments were post-fixed with 1% osmium tetroxide for 30 min, and stained with 2% uranyl acetate for 1 h, and dehydrated with a rising ethanol series (50-70-80-90-96-100-100%), followed by critical-point drying. Dried specimens were mounted on adhesive carbon tape and sputter-coated with 10 nm Au using Bal-Tec SCD 050. Samples were imaged with a Zeiss Sigma 300 VP (Zeiss) using 1 kV accelerating voltage and a secondary electron detector. For transmission electron microscopy (TEM), fixed samples were cut into 100 μm thick sections using a vibratome Zeiss Hyrax V50 (Zeiss). Sections were post-fixed with 1% osmium tetroxide for 1 h, stained with 2% aqueous uranyl acetate for 1 h, and dehydrated using a graded ethanol series (50-70-80-90-96-100-100%), each step being 15–30 min, followed by acetone (100%) 2× 30 min. Samples were infiltrated with an increasing concentration of epon resin or Spurr low-viscosity resin (both Sigma-Aldrich, St. Louis, MI, USA) (33-66-100%) over three days. Finally, segments were flat-embedded between PTFE-coated glass slides and heat-polymerized at 65 °C for 48 h. After re-mounting, sections were cut using a Leica UC7 ultramicrotome (Leica) and Diatome diamond knives (Diatome, Quakertown, PA, USA). Semi-thin, 1 μm epon sections were transferred onto adhesion slides Superfrost^®^ Plus, stained with Richardson’s solution (alkaline solution of azure II and *methylene blue*), and imaged with a Zeiss Primostar upright microscope equipped with an Axiocam 105 color camera and ZEN 3.2 software, using 10× and 40× Plan-Achromat objectives. Ultra-thin, 80 nm sections were deposited on copper, slot, formvar-coated grids, and contrasted with saturated aqueous uranyl acetate for 10 min, followed by Reynolds lead citrate for 3 min. Grids were imaged with a CM10 transmission electron microscope (Philips, Amsterdam, The Netherlands), operated at 80 kV, and equipped with an LaB6 filament, a CCD side-mounted MegaView III camera (Emsis, Berlin, Germany), and iTEM software version 9 (both Olympus Soft Imaging solutions, Munster, Germany).

### 2.5. Determination of the Root Growth and the Extent of Infection

For the root growth analyses, *B. distachyon* germlings were grown vertically on a solid VMM medium with 0.2% sucrose for 7 days at 22 °C and 16 h/8 h long day conditions. After 7 days, half of the germlings were inoculated with *N. crassa* macro-conidiospores. Uninoculated germlings were grown as negative controls under the same conditions. Root length (mm) was determined daily for 140 h post-inoculation. The means and standard deviations of three biological replicates were determined. Significant differences between non-treated and treated root lengths were determined by a T-test analysis. Significance is indicated as * (* *p* < 0.1, ** *p* < 0.05, *** *p* < 0.005).

For investigating the extent of the infection of the *B. distachyon* root vascular bundle by *N. crassa*, paraffin sections were prepared as described above. In total, nine plants were analyzed. For each plant sample, the extent of infection was evaluated on serial paraffin sections through app. 1 mm long root segments collected up to 1 cm away from the root tip. A total of 2–4 segments were analyzed for each plant sample. The score was assigned on a scale from 0 (no hypha) to 4 (extensive growth of hypha). The mean value and the standard deviation were calculated by Excel 2024 (according to the formula AVERAGE and STDEV.S, respectively).

## 3. Results

In 2014, a study indicated an endophytic lifestyle of *N. crassa* in pine trees [15]. However, despite our vigorous attempts, we could not establish an interaction between Pinus roots and the fungus—further efforts with *Arabidopsis thaliana* and *Zea mays* also remained unsuccessful (Figure S1). In the case of Pinus seedlings, extensive fungal growth could be detected surrounding the roots and the interaction was stable in the sense that the seedlings survived the co-culturing with *Neurospora crassa*; however, we were unable to identify any fungal growth inside the plant by microscopic investigations (Figure S1A). Both *A. thaliana* and *Z. mays* seedlings were unable to survive the co-cultivation with *N. crassa*. A few days after inoculation, the plants developed necrotic symptoms visible especially on the leaves and were rapidly overgrown and saprotrophically digested by the fungus. In the early stages after inoculation, before the saprotrophic switch, hyphae could not be observed inside the plant tissue (Figure S1B,C). Finally, we employed the model grass *Brachypodium distachyon*, which is believed to interact with numerous microbes present in the soil [35].

**Figure 1:**
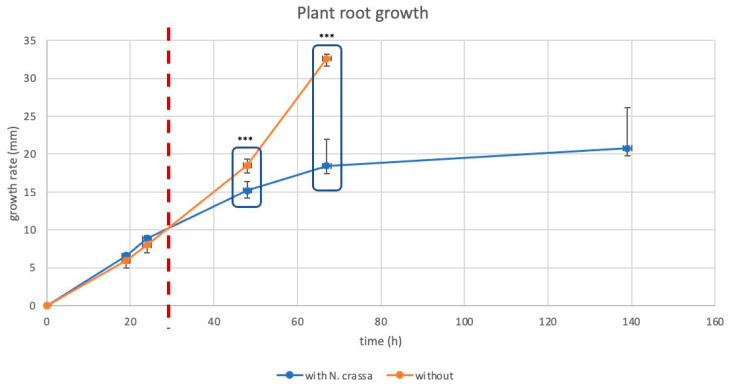
Growth of *B. distachyon* roots with (blue) and without (orange) *N. crassa* co-cultivation. The red dashed line represents the time point, at which *N. crassa* spatially reaches the roots in the co-cultivation experiment. Significant differences between roots in the same developmental stage (grouped by boxes) are depicted as *** (*p* < 0.005).

Fungal colonization by *N. crassa* of *B. distachyon* followed the same pattern in all experiments: We observed extensive growth of *N. crassa* around the plant root, while the *B. distachyon* shoot did not seem to be affected (Figure S2). Co-culturing of *N. crassa* with *B. distachyon* led to a significant reduction in root growth after 48 h (*p* = 3.134 × 10^−5^), an effect which was even stronger after 67 h with root growth stopping after 139 h (Figure 1).

**Figure 2:**
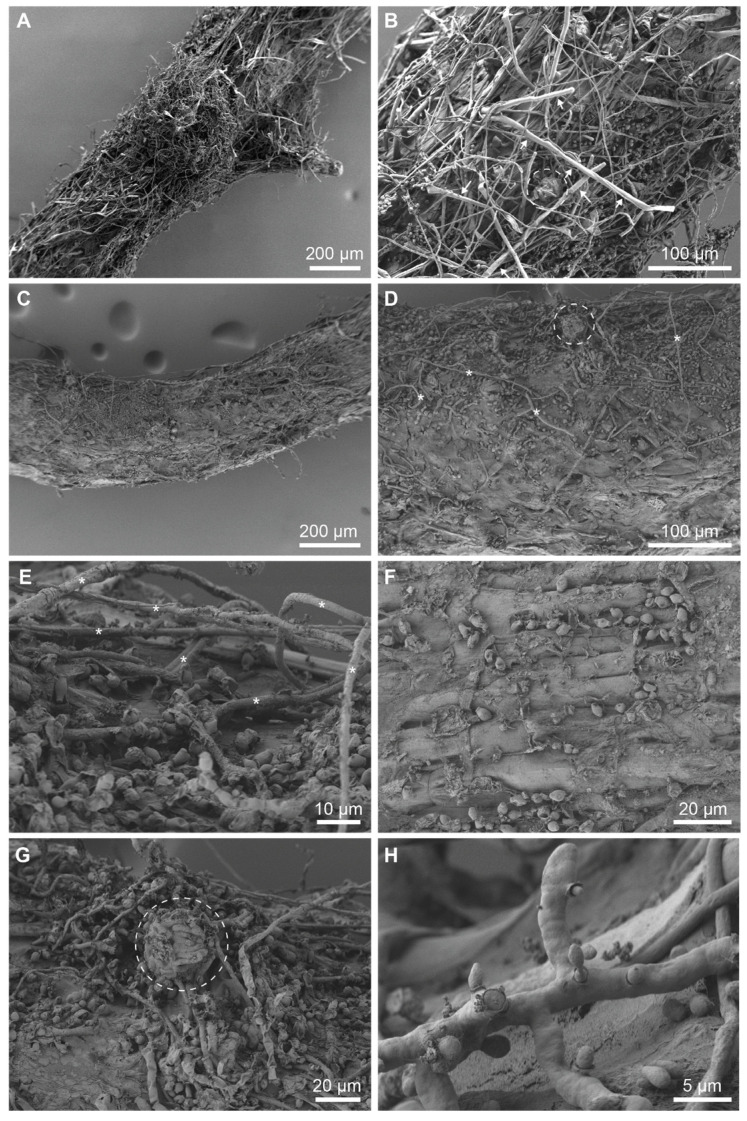
The SEM visualization of *B. distachyon* roots infected with *N. crassa*. Roots are wrapped in a dense network of hyphae (**A**–**D**) and scattered with oval-shaped conidia (**C**–**H**). Hyphae of different thickness can be observed (**E**). The integrity of the root surface is preserved (**F**). Protoperithecia (encircled) form in the vicinity of a root (**B**,**D**,**G**). The Microconidia production of old hyphae (**H**). Asterisk (*): hypha; circle: protoperithecium; white arrow: root hair.

Using the *N. crassa* Histon1:GFP line (#9518), which allows for the easy recognition of fungal hyphae, we observed extensive fungal growth around the plant root and an attachment of hyphae to the root surface. Moreover, there was a strong formation of protoperithecia in the root vicinity (Figure S3). Scanning electron microscopic images of infected roots substantiated our previous observations revealing an intact root epidermis with a tightly surrounding mycelial network (Figure 2).

**Figure 3:**
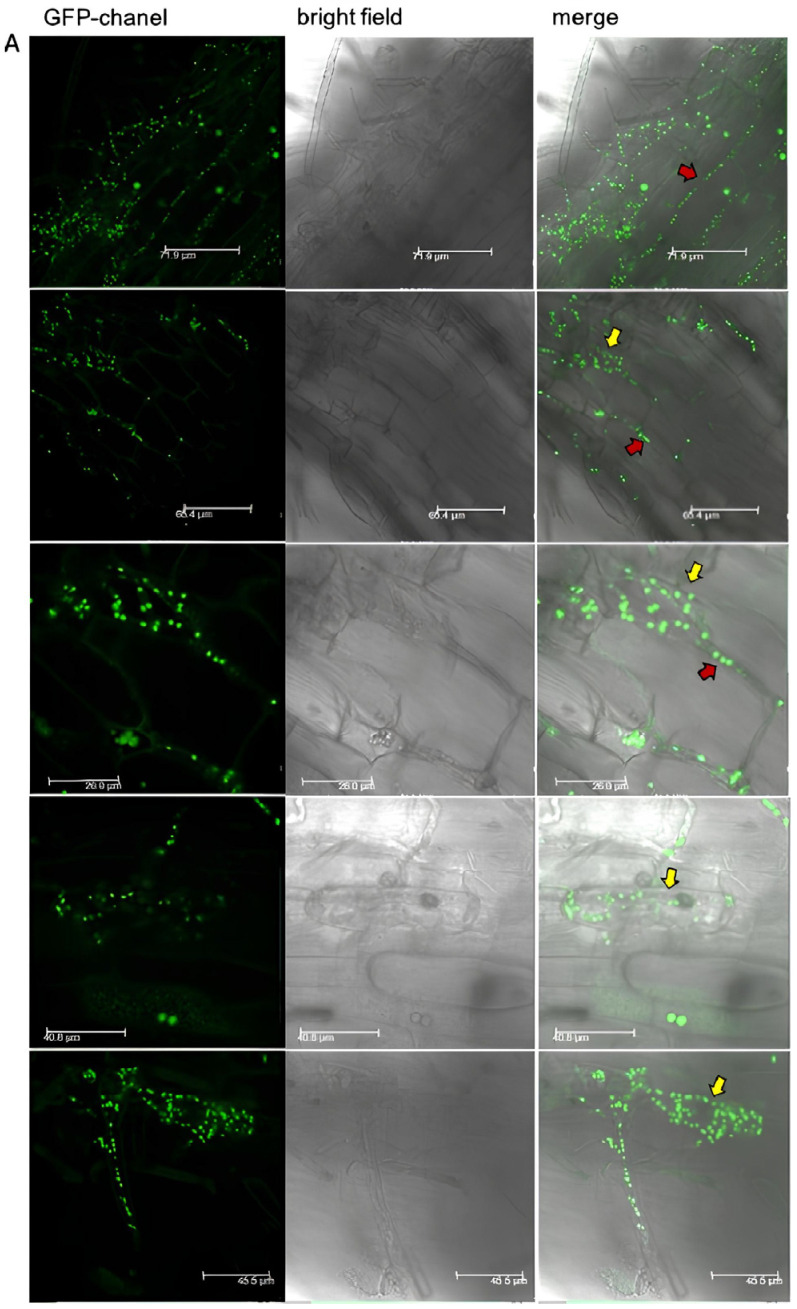

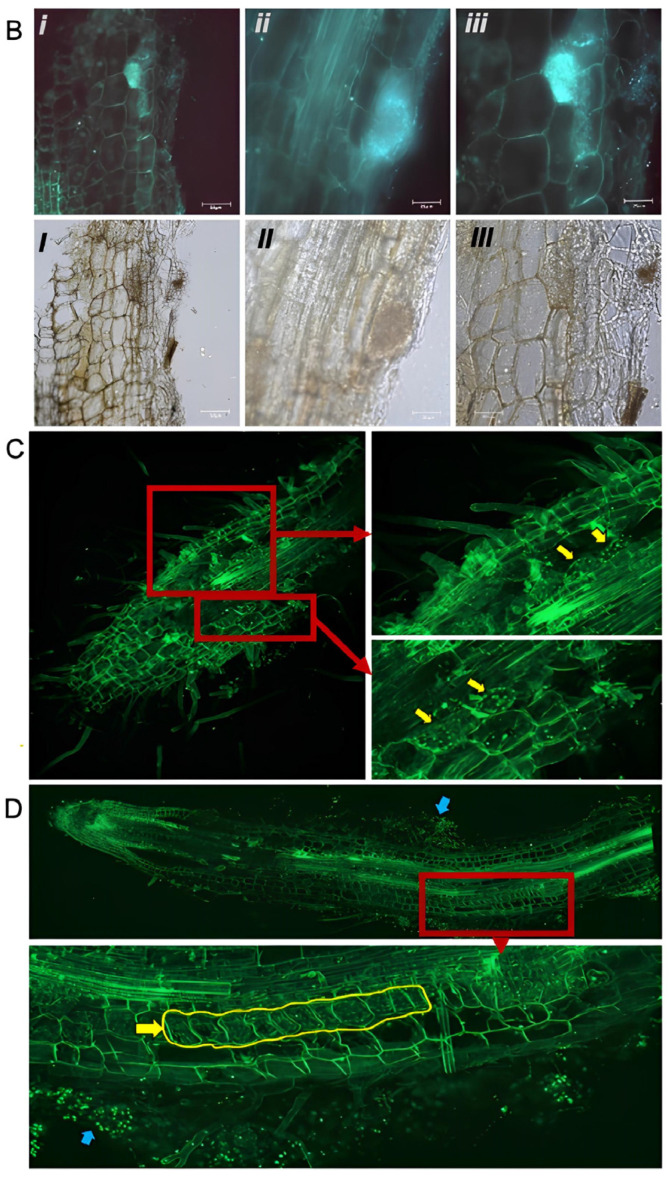
Fluorescent images of longitudinal *B. distachyon* root sections co-cultivated with the *N. crassa* FGSC #9518 strain, expressing GFP-coupled histone1 protein. Additional green fluorescence results from the auto-fluorescing plant cell wall. (**A**) Confocal laser scanning images show apoplastic (red arrows) and subcellular (yellow arrows) growth of fungal hyphae. (**B**) Fluorescent microscopic images show *N. crassa* accumulation in single cortical plant cells. (***i–iii***) GFP channel, (***I–III***) corresponding brightfield images. (**C**,**D**) Spinning-disk confocal microscopy was used to present an overview of a larger root section area. Distinct sectors (depicted by red boxes) with invasive *N. crassa* growth (yellow arrows) are magnified. Blue arrows indicate the extensive hyphal network covering the root surface.

**Figure 4:**
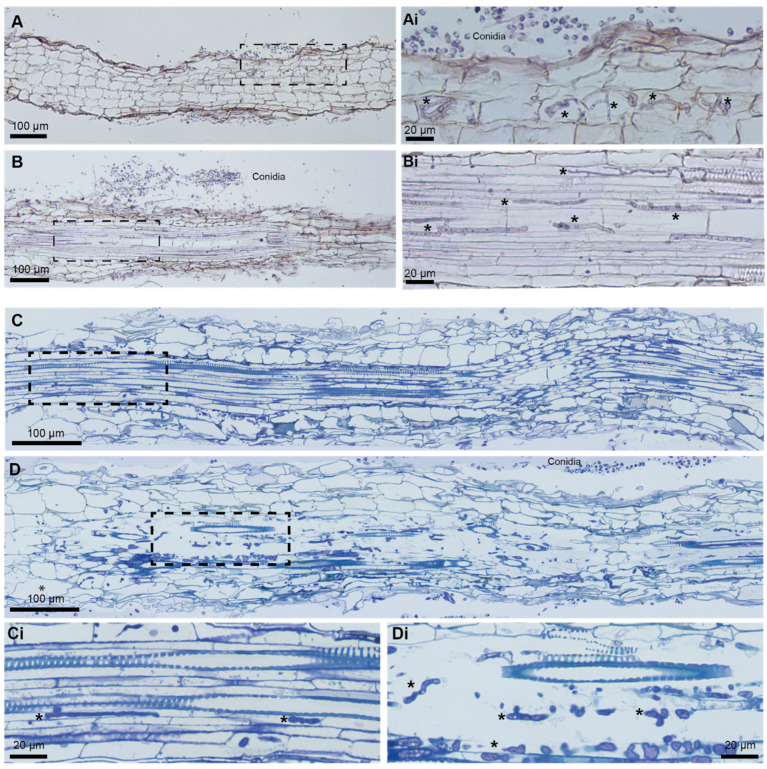
The histological analysis of *B. distachyon* roots infected with *N. crassa*. (**A**,**B**) Paraffin sections, 7 μm, stained with hematoxylin and eosin. Conidia outside the root (**A**,**B**). Hyphae (*) seen inside a few plant root cortical cells whereas the majority of root cortical cells do not appear to be infected (**A**). Hyphae (*) growing through the vascular bundle (**B**). (**C**,**D**) Epon sections, 1 μm, stained with Richardson’s solution. Hyphae (*) growing inside the vascular bundle of a moderately infected root with vascular elements preserved (**C**), and of a strongly infected root with disrupted vascular elements (**D**). Xylem elements can be recognized by secondary wall thickenings. Panels (**i**) display details of boxed areas. Asterisk (*): hypha.

**Figure 5:**
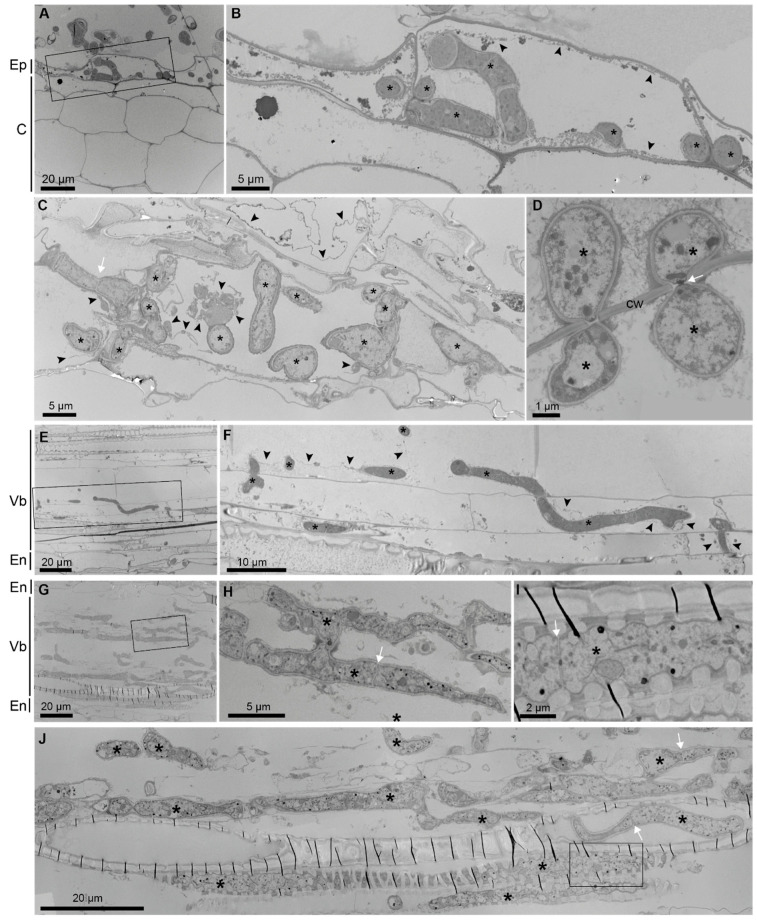
The TEM analysis of *B. distachyon* roots infected with *N. crassa*. Hyphae in epidermal cells (**A**,**B**), in cortical cells (**C**,**D**), in a moderately infected vascular bundle (**E**,**F**), and in a strongly infected vascular bundle (**G**–**J**). The plant cell cytoplasm/membrane (arrowheads) can be observed in infected cells and neighboring cells (**C**). Hyphae spread through the root by crossing plant cell walls (**D**,**F**). In a moderately infected root, plant vascular elements are largely preserved (**E**,**F**). In a strongly infected root, vascular elements are destroyed except for the xylem elements with secondary wall thickenings (**G**,**J**). Parallel, thin, black lines represent folds that were generated over these rigid cell walls during sectioning (**G**,**J**). Hyphae grow unrestricted (**H**) or inside the xylem vascular elements (**I**,**J**). The characteristic ultrastructure of fungal hyphae (*) includes the cell wall, septa (with pores) (arrows), and dense cytoplasm. Boxed areas (**A**,**E**,**G**,**J)** are shown as enlarged (**B**,**F**,**H**,**I**). Asterisk (*): hypha; white arrow: hyphal septum; black arrowhead: plant cell cytoplasm/membrane; Ep: epidermis; C: cortex; En: endodermis; Vb: vascular bundle; Cw: cell wall.

The strong effect of the fungus on the plant root let us further investigate the interaction of *N. crassa* and *B. distachyon*. We collected roots with an attached fungal network, which were then prepared for an on-section analysis by confocal laser scanning (CLSM, Figure 3 and Figure 4) and transmission electron microscopy (TEM, Figure 5). Interestingly, we observed fungal hyphae within the root, not only growing in the intercellular space but also in some of the root cortex cells and inside the vascular bundle.

Cross- and longitudinal sections imaged by CLSM showed overall living root tissues, mostly unimpaired by fungal invasion. However, single hyphae showed a clear growth in the apoplastic space (Figure 3 red arrows), and in single root cortex cells, an accumulation of fungal hyphae was visible (Figure 3 yellow arrows). Those invaded cells were in most cases separated and surrounded by non-infected, living neighbor cells; however, in some cases, a whole cell layer of cortical cells showed fungal accumulation in a very similar manner (Figure 3D). We could not observe the formation of an extensive Hartig-net-like hyphal network in any case [36]. Paraffin sections and semi-thin resin sections similarly indicated a limited invasion of the cortical tissues (Figure 4). In addition, fungal hyphae were more readily detected inside the vascular bundle. In a few cases of extensive fungal growth inside the vascular cylinder, the cortical tissues appeared to be notably less affected. To quantify the colonization rate of the vascular bundle by *N. crassa* in different *B. distachyon* roots, serial paraffin sections through app. 1 mm long root segments collected up to 1 cm away from the root tip were evaluated and assigned a score of 0 to 4 (0 (no hypha); 4 (extensive growth of hypha)). As shown in Figure S4, except for one plant, all investigated plants showed the colonization of the vascular bundle. However, the extent of colonization was generally rather low with both median (0.88) and standard deviation (1.268) values below 2 (defined as the middle of the colonization scale).

TEM images provided additional detailed information about the distribution and spreading of fungal hyphae in the plant tissues (Figure 5). In the ultra-thin sections, fungal hyphae could be detected in every layer of the plant root from the epidermis to the xylem elements of the vascular bundle. As shown in Figure 5D,F, fungal hyphae cross the plant cell wall and enter neighboring cells over the plasmodesmata. In the case of extensive growth inside the vascular bundle, plant cells seemed largely destroyed except for xylem elements with secondary cell walls (Figure 5G–J).

Our data demonstrate that *N. crassa* exhibits an endophytic growth pattern in *B. distachyon*.

## 4. Discussion

*Neurospora crassa* is a model organism that is widely used in research, even though little is known about its natural lifestyle. Wild Neurospora strains are usually collected from burned vegetation, where the genus appears as the first fungal colonizer, probably germinating from single ascospores [12,13,14]. Nevertheless, most of the strains collected belong to *N. intermedia*. Pandit and Maheshwari conducted the only ecological study on an indigenous Neurospora species [37,38]. In a sugarcane field that was burned periodically, they examined a population of *N. intermedia*. Even though Neurospora can be cultivated from soil samples, visible and excessive growth only occurs when vegetation has been exposed to fire. The persistence of Neurospora mycelia or spores between two fires remains a mystery. One study suggested that the natural habitat of *N. crassa* is inside pine trees, where the fungus shows a varied lifestyle, from mutualistic to pathogenic depending on the growth conditions [34].

Although there is no direct evidence that *N. crassa* infects *B. distachyon* in the wild, *B. distachyon*’s geographical distribution partially overlaps that of *N. crassa* and the two species favor similar climatic conditions. Sordariales fungi other than *N. crassa* dominate the rhizosphere microbiome of *B. distachyon* [35]. There are, however, records of *N. crassa* occurring on different grass species, including wild and cultivated Saccharum [12], which is closely related to *B. distachyon*. Our study shows *N. crassa* developing an endophytic interaction with *B. distachyon*, which may indicate that the fungus is naturally associated with Poaceae. The fungal interaction with *B. distachyon* does not affect its fitness in the early stages of interaction, contrary to our investigation of other plant species where *N. crassa* rapidly overgrew and eliminated the plant counterpart. Although root growth was significantly inhibited, leaf and shoot development was similar to that of non-infected plants, suggesting that the water supply was normal. *N. crassa*, however, switches from a pathogenic or saprotrophic lifestyle when the balance of the interaction is disturbed. Such a lifestyle versatility is characteristic of endophytic fungi [39,40]. Nonetheless, since endophytic fungal interactions are usually beneficial to plants [41], it remains unclear whether *N*. *crassa* impacts plant development in any positive way. The results of our study also hint towards *N. crassa* in the wild being associated with the Poaceae family. The collection of wild isolates from the grass rhizosphere may be a promising approach to confirm this hypothesis.

Crossing experiments with *N. crassa* in the laboratory are standard, but *N. crassa*’s sexual development in nature remains an enigma. During the present study, extensive protoperithecia formation was observed on the plant roots. The high nitrogen consumption of Poaceae could explain this phenomenon by depleting nitrogen around roots, promoting early sexual development of the fungus [42,43,44]. To determine whether protoperithecia can mature in the interaction system, further studies will be conducted.

Our study, together with the results investigated in pine trees [15], demonstrates the ability of *N. crassa* to switch from a saprotrophic to an endophytic lifestyle. This phenomenon is restricted to distinct plant species, as our attempts with *N. crassa* to establish an enduring endophytic interaction failed with other mono- and dicotyledons. Several fungi display a dual niche, which means that they can switch between different lifestyles depending on the interacting species [45,46,47]. Our data support the emerging evidence that the capability of fungi to colonize multiple ecological niches is a general and common phenomenon. Which interaction a fungus and a plant develops depends on a variety of factors, in a. o. the genotypes of the interacting species, the plant microbiome, nutrient supply, or stressors (reviewed in [26]). Especially rapidly changing climate conditions can disturb stable, beneficial plant–fungus interactions, forcing a saprotrophic or phytopathogenic switch in the fungal life cycle, and causing severe damage in natural and agricultural systems [48,49]. Several studies reported the beneficial effect of EF on plant fitness and presented their potential agricultural applications (in a. o. [20,23,39,50,51,52]). However, a better understanding of the establishment of endophytic fungal interactions on the molecular level is needed, so that we will be able to develop customized, powerful, and harmless solutions for natural and agricultural systems. Our system, which combines two well-established model organisms for genetical, molecular, and cell biological studies, provides a powerful tool for studying the general properties of plant–fungus interactions on the molecular level.

## Data Availability

All presented data are available at Mendeley Data. DOI:10.17632/bzj5zv36kw.2.

## Supplementary Materials

The following supporting information can be downloaded at: https://www.mdpi.com/article/10.3390/jof10070487/s1, Figure S1: Co-cultivation of *N. crassa* with Pinus, *A. thaliana* and *Zea mays.* Figure S2: Co-cultivation of *B. distachyon* and *N. crassa* in an EcoFab device with fluid medium (A) and on solid medium in Petri dishes (B) and falcon tubes (C). Figure S3: Extensive proto-perithecia formation of *N. crassa* FGSC #9518 strain in co-cultivation experiments with *B. distachyon*. Figure S4: Extent of vascular bundle colonization of different *B. distachyon* roots by *N. crassa*.
