# Supplementary material for: The First Observation of the Filamentous Fungus Neurospora crassa Growing in the Roots of the Grass Brachypodium distachyon
Source: J Fungi (Basel). 2024 Jul 14;10(7):487. doi: 10.3390/jof10070487 (PMC11278470; doi:10.3390/jof10070487)
Supplement: Supplementary file 1 [file jof-10-00487-s001.zip › jof-3079855-supplementary.pdf]

SUPPLEMENTARY FIGURES

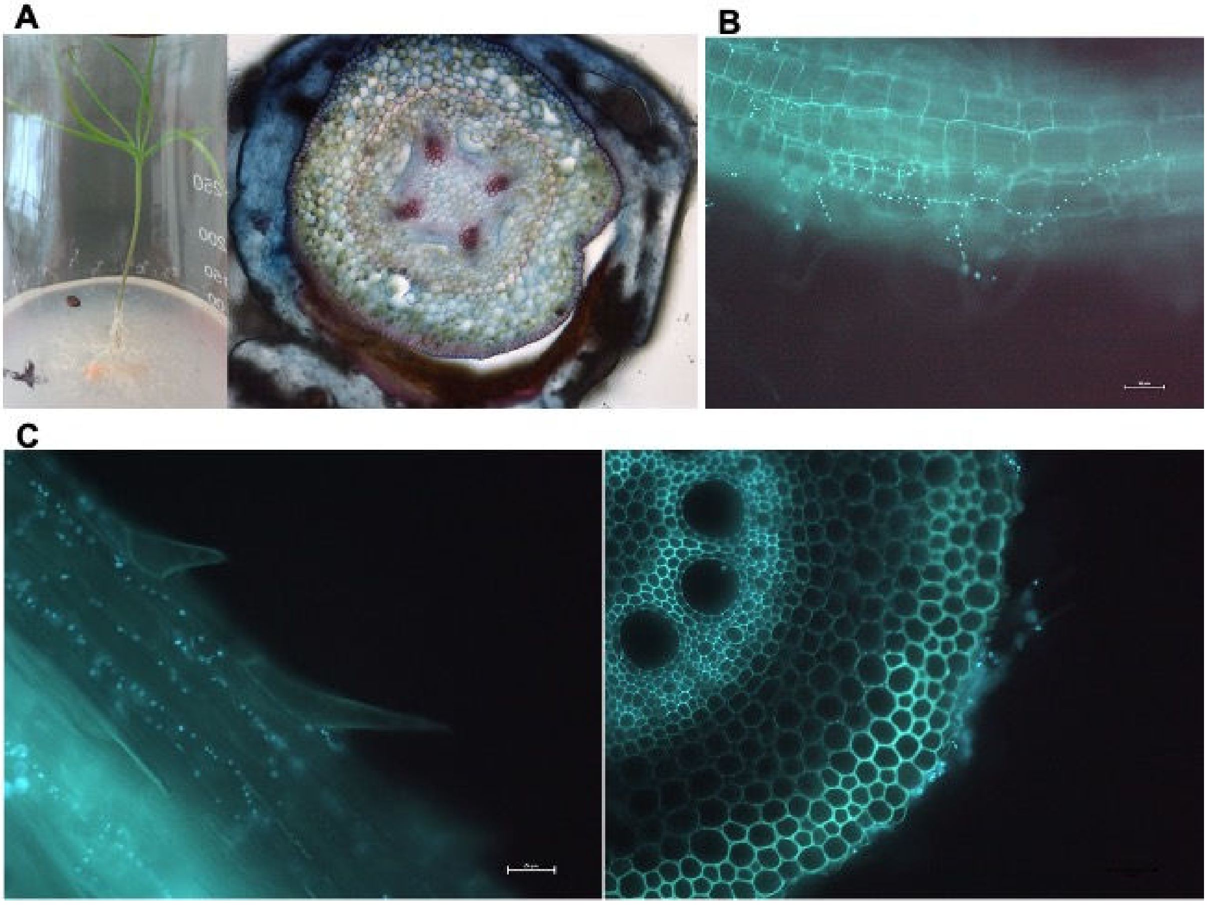

Figure S1: Co-cultivation of *N. crassa* with Pinus (**A**), *A. thaliana* (**B**) and *Zea mays* (**C**). In the experiments with Pinus a wildtype *N. crassa* (FGSC #4200) strain was used. The cross-section of the root was stained with FCA-solution. The extensive hyphal mantel surrounding the root has no obvious connection to the plant rhizodermis. No fungi could be detected inside of the roots. In the experiments with *A. thaliana* and *Z. mays*, an *N. crassa* strain expressing gfp-coupled histone (resulting in green fluorescent nuclei within the hyphae) was used to recognize fungal hyphae in the plant tissue easily. Fluorescent images of *A. thaliana* and *Z. mays* show single hyphae on the root surface (**B, C**). No fungal growth could be detected in root-sections (**C**, picture on the right).

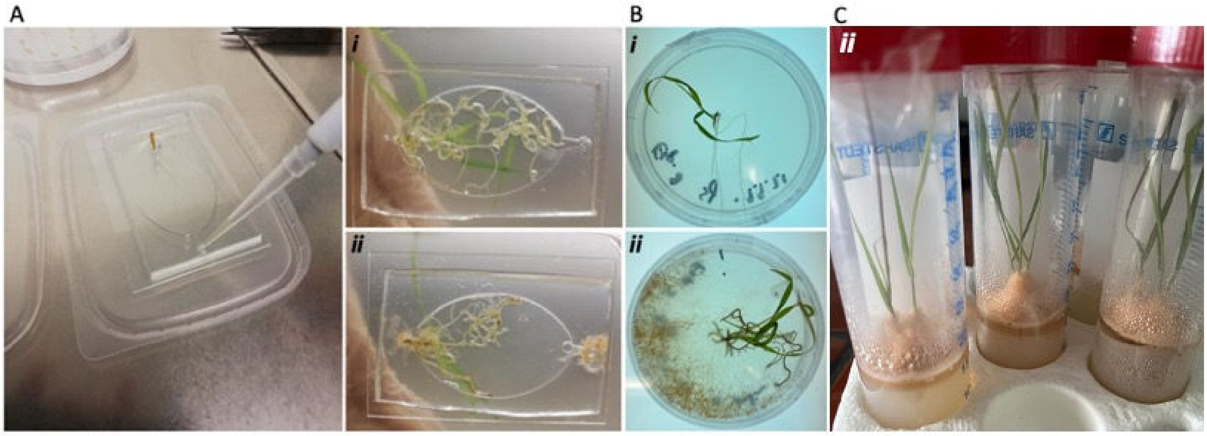

Figure S2: Co-cultivation of *B. distachyon* and *N. crassa* in an EcoFab device with fluid medium (**A**) and on solid medium in Petri dishes (**B**) and falcon tubes (**C**). Uninfected plants (***i***), infected plants (***ii***)

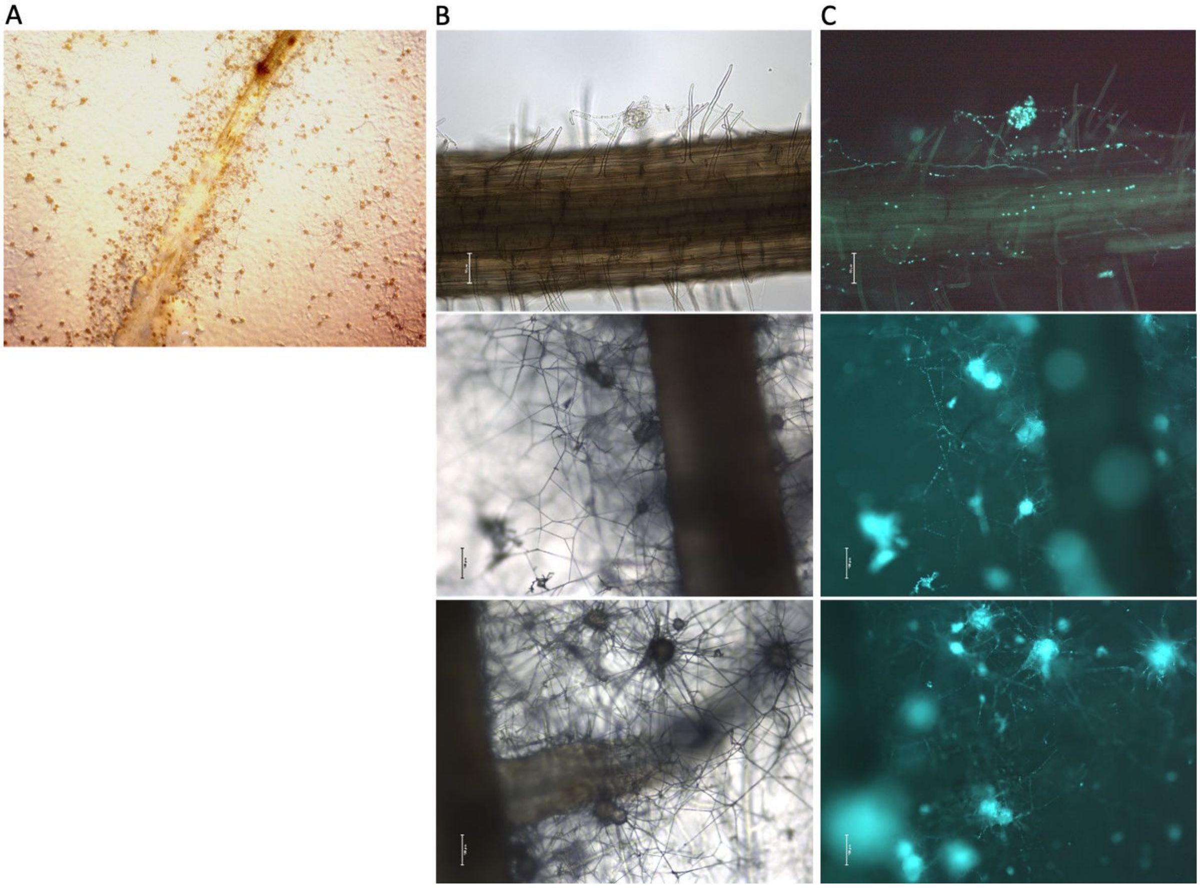

Figure S3: Extensive proto-perithecia formation of *N. crassa* FGSC #9518 strain in co-cultivation experiments with *B. distachyon*. (**A**) Stereo microscopic image of an infected root at the root hair zone with several proto-perithecia (small spots). (**B**) Bright-field and (**C**) fluorescent images of the hyphal network with several proto-perithecia surrounding the plant root.

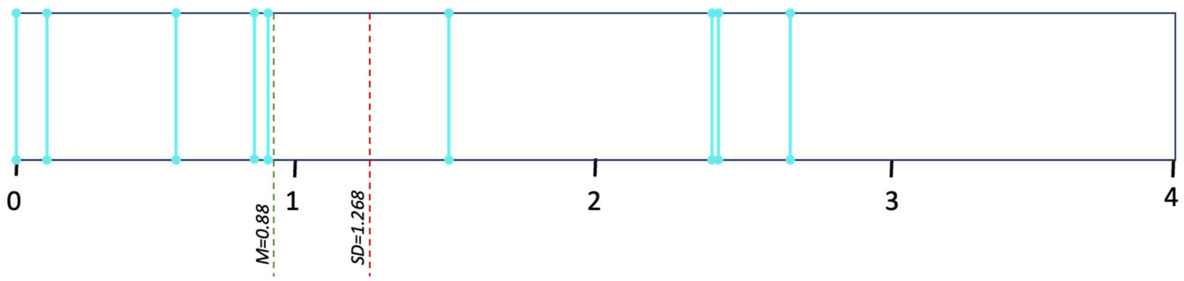

Figure S4: Extent of vascular bundle colonization of different *B. distachyon* roots by *N. crassa*. Blue lines represent individual plant samples. For each plant sample, the extent of infection was evaluated on serial paraffin sections through app. 1 mm long root segments collected up to 1 cm away from the root tip. Two to four segments were analyzed for each plant sample. The score was assigned on a scale from 0 (no hypha) through 4 (extensive growth of hypha). M: median; SD: standard deviation
